# Involvement of miR-205-5p in mediating the development of nodular thyroid disease associated with coal worker’s pneumoconiosis via pulmonary extracellular vesicles

**DOI:** 10.3389/fendo.2025.1528330

**Published:** 2025-07-04

**Authors:** Feng Zhao, Yu Hao, Hongzhen Zhang

**Affiliations:** ^1^ Thyroid and Breast Surgery Department, The First Hospital of Anhui University of Science and Technology, Huainan First People’s Hospital, Huainan, China; ^2^ Breast Surgery, Provincial Hospital of Weihai City, Weihai, Shandong, China; ^3^ School of Public Health, Anhui University of Science and Technology, Huainan, China

**Keywords:** pulmonary extracellular vesicles, MiR-205-5p, coal worker’s pneumoconiosis, nodular thyroid disease, ATF4/CHOP signaling axis

## Abstract

**Objective:**

Coal worker’s pneumoconiosis (CWP) is an occupational disease, and the mechanisms underlying the development of its complication, nodular thyroid disease (NTD), remain unclear. This study aimed to investigate the role of miR-205-5p (miR-205-5p) in the development of nodular thyroid disease associated with coal worker’s pneumoconiosis.

**Methods:**

The potential role of pulmonary extracellular vesicles in triggering coal worker’s pneumoconiosis-associated nodular thyroid disease was explored. RNA was extracted from isolated extracellular vesicle samples, and real-time fluorescent quantitative RT-qPCR was performed using specific primers and probes. The levels of miR-205-5p in the extracellular vesicles from the supernatant of each treatment group were compared, and the significant expression of miR-205-5p in the extracellular vesicles was detected by RT-qPCR to evaluate the regulatory role of lung-derived extracellular vesicles in the development of coal worker’s pneumoconiosis-associated nodular thyroid disease. In addition, cell proliferation, apoptosis, and invasion capabilities were assessed using the CCK-8 assay for cell proliferation activity, Annexin V-FITC/PI double staining and flow cytometry for cell apoptosis rate, and Transwell assay for cell invasion ability.

**Results:**

By isolating, purifying, and analyzing extracellular vesicles from coal worker’s pneumoconiosis patients and healthy controls, it was found that the expression of miR-205-5p in the plasma of coal worker’s pneumoconiosis patients was significantly higher than that in the healthy controls (*P*<0.01). Furthermore, the expression levels of ATF4 and CHOP were also significantly increased in coal worker’s pneumoconiosis patients (*P*<0.01), consistent with the expression trend of miR-205-5p. Further *in vitro* cell experiments demonstrated that miR-205-5p can promote the proliferation and formation of nodules in thyroid cells by regulating the expression of downstream target genes. This study revealed the critical role of miR-205-5p in the development of nodular thyroid disease associated with coal worker’s pneumoconiosis and provided new experimental evidence for the diagnosis and treatment of this disease.

**Conclusion:**

miR-205-5p in pulmonary extracellular vesicles mediates the development of nodular thyroid disease associated with coal worker’s pneumoconiosis through the ATF4/CHOP signaling axis. Further research is essential to comprehensively investigate the specific targets through which miR-205-5p derived from pulmonary extracellular vesicles triggers the development of nodular thyroid disease associated with coal worker’s pneumoconiosis via the ATF4/CHOP signaling axis.

## 1 Introduction

Coal worker’s pneumoconiosis (CWP) is a severe occupational disease primarily affecting workers with prolonged exposure to coal dust (1–5). CWP not only impairs lung function but also potentially leads to a range of complications, including nodular thyroid disease (NTD) (6). In recent years, the role of pulmonary extracellular vesicles in disease progression has garnered increasing attention (7). Extracellular vesicles are tiny vesicles capable of carrying various bioactive molecules, such as proteins, lipids, and nucleic acids, facilitating intercellular communication. Research has shown that extracellular vesicles play significant roles in numerous diseases, emerging as a promising field for studying disease mechanisms and diagnostic biomarkers (8–10).

In the study of CWP-associated NTD, microRNAs (miRNAs) carried by extracellular vesicles are of particular interest. miRNAs are small RNA molecules, 18–25 nucleotides in length, that regulate gene expression by binding to the mRNA of target genes. It has been found that miRNAs in extracellular vesicles are crucial in the onset and progression of various diseases. miR-205-5p, as one of these miRNAs, has been demonstrated to have regulatory functions in several cancers (11). However, its role in CWP-associated NTD remains unclear.

This study focuses on whether pulmonary extracellular vesicles in coal worker’s pneumoconiosis (CWP) patients contain miR-205-5p and whether it influences the development of nodular thyroid disease (NTD) by regulating the ATF4/CHOP signaling axis. ATF4 and CHOP are critical molecules involved in cellular stress responses and play key roles in regulating various cellular functions. Studies have shown that the ATF4/CHOP signaling axis is significant in apoptosis and inflammatory responses, and is closely related to the pathological processes of various diseases.

The significance of this research lies in uncovering the mechanism of action of miR-205-5p in pulmonary extracellular vesicles from CWP cases, thereby providing new molecular targets for the early diagnosis and treatment of CWP-associated NTD. By thoroughly investigating the relationship between miR-205-5p and the ATF4/CHOP signaling axis, this study aims to offer theoretical foundations and experimental data to support clinical prevention and treatment strategies for CWP and its complications. Additionally, this research could expand the application of extracellular vesicle studies in the field of occupational diseases, serving as a reference for research into other related diseases.

## 2 Materials and methods

### 2.1 Sample collection

We selected 30 patients diagnosed with coal worker’s pneumoconiosis (CWP) from January 2023 to January 2024 at the Huaihe Energy Group Coal Miners Hospital in Anhui Province. The patients are aged between 45 and 65 and all have a history of working in underground coal mines for more than ten years. Additionally, we recruited a control group of 30 healthy individuals, matched for age and gender with the patient group. All participants had no prior history of other chronic diseases, and those with a history of nodular thyroid disease, diabetes, or cardiovascular diseases were excluded. All patients provided informed consent. Plasma samples and bronchoalveolar lavage fluid (BAL) were collected from these participants. We also used the 16HBE cell line for *in vitro* experiments. The samples used in this study were all collected from directly recruited patients with coal workers’ pneumoconiosis (CWP) and healthy control individuals. The collection of these samples strictly followed the approval of the Ethics Committee of the First Affiliated Hospital of Anhui University of Science and Technology (the First People’s Hospital of Huainan City) (Ethical Approval Number: 2022-YJ-020-01), and the informed consent of all participants was obtained. To ensure the representativeness of the samples and the clinical relevance of the conclusions, we specially selected healthy control individuals whose age and gender were matched with the patient group, and all participants had no history of other chronic diseases. Moreover, the sampling was conducted by professional technicians to ensure the standardization and consistency of sample collection.

### 2.2 Extraction of pulmonary extracellular vesicles

Extracellular vesicles were isolated from the culture supernatant of 16HBE cells using ultracentrifugation. The specific steps included centrifuging the culture supernatant at 300g for 10 minutes to remove cell debris and large particles; followed by centrifugation at 2000g for 20 minutes to remove larger cellular debris; subsequently, centrifugation at 10,000g for 30 minutes to eliminate smaller particles and debris; finally, ultracentrifugation at 100,000g for 70 minutes to collect the extracellular vesicle pellet. The extracellular vesicle pellet was resuspended in PBS solution and stored at -80°C for future use.

### 2.3 Cell experiment design

The experiment utilized the 16HBE cell line, a bronchial epithelial cell line commonly used in pulmonary disease research. Four groups were established: a control group, a miR-205-5p overexpression group, a miR-205-5p inhibition group, and an empty vector group. The control group received no treatment, serving as a baseline comparison. The miR-205-5p overexpression group was transfected with miR-205-5p mimics to increase the level of miR-205-5p. The miR-205-5p inhibition group was transfected with miR-205-5p inhibitors to reduce its expression level. The empty vector group was transfected with an inert vector, serving as a negative control for the experiments.

### 2.4 Cell transfection

Cell transfection was performed using Lipofectamine 3000 reagent. First, 16HBE cells were cultured to the logarithmic growth phase, reaching a cell density of 70%-80%. According to the manufacturer’s instructions, miR-205-5p mimics, inhibitors, and the empty vector were each mixed with Lipofectamine 3000 and incubated at room temperature for 5 minutes before being added to the cell culture dishes. After 6 hours of transfection, the medium was replaced with fresh culture medium, and the cells were incubated for an additional 24 hours to ensure transfection efficiency.

### 2.5 Quantitative PCR detection

Quantitative PCR (q-PCR) technology was employed to detect the expression levels of miR-205-5p, ATF4, and CHOP in the plasma samples of CWP cases. Plasma samples were collected from CWP cases and healthy controls, and RNA extraction was performed immediately to ensure RNA integrity and quality. Total RNA was extracted using Trizol reagent, and mRNA was converted to cDNA via reverse transcription. For accurate results, specific primers and probes were used, and SYBR Green fluorescent dye was added to the q-PCR reaction system to monitor the accumulation of amplification products in real-time. Specific primers for miR-205-5p, ATF4, and CHOP were employed, with U6 and GAPDH serving as internal reference genes for normalization. Relative expression levels were calculated using the standard curve method. A list of primer sequences is provided in **Supplementary Material 2**.

### 2.6 Western Blot analysis

This study employed Western Blot (WB) technology to analyze the expression of proteins in tissue samples. Approximately 50 mg of tissue sample was retrieved from a -80°C freezer, minced, and placed in a sterile glass homogenizer. A mixture of PMSF, cell lysis buffer, and protease inhibitors (0.1g/1ml) was added, and the samples were homogenized on ice for 5 minutes. The homogenate supernatant was transferred to a 1.5 ml EP tube and centrifuged at 12,000 g for 20 minutes at 4°C after a 30-minute ice bath. The supernatant was used for WB experiments or stored at -80°C.

Protein quantification was performed using the BCA method. The BCA reagent was mixed in a 50:1 ratio, and the standard was diluted to 0.5 mg/ml before being added to a 96-well plate. After supplementing the sample to 20μl,200 μl of BCA working solution was added, followed by a 30-minute incubation at 37°C. Absorbance was measured at 562 nm, a standard curve was plotted, and protein concentration was calculated. Finally, 5× loading buffer was added, and the sample was heated for 10 minutes to complete preparation. Subsequently, 12% resolving gel and 5% stacking gel were prepared for electrophoresis. After transferring proteins to a PVDF membrane, the membrane was activated in methanol and assembled into a “transfer sandwich” for constant voltage transfer. The membrane was washed with TBST for 2 minutes, blocked quickly for 30 minutes, and incubated with primary antibodies overnight at 4°C, followed by three washes. Secondary antibody incubation was conducted at room temperature for 2 hours, with three more washes. Finally, the PVDF membrane was exposed to chemiluminescent reagent for 3 minutes, and protein bands were detected using a chemiluminescence imaging system. ImageJ software was used to analyze the optical density values, calculating the relative expression of target proteins compared to internal control proteins. Antibody dilution ratios are detailed in **Supplementary Material 3**.

### 2.7 Immunofluorescence detection

Immunofluorescence technology was utilized to detect the expression of ATF4 and CHOP proteins in BAL samples from different patient groups, including a healthy control group, a CWP patient group, and a CWP patient group with nodular thyroid disease (NTD). BAL samples were first centrifuged, and cells were resuspended in PBS. They were then placed on slides treated with poly-L-lysine. The samples were fixed with 4% paraformaldehyde, incubated at room temperature for 10 minutes, and washed. Permeabilization was conducted using 0.1% Triton X-100, followed by another wash after a 10-minute incubation at room temperature. Blocking was performed with PBS containing 5% bovine serum albumin (BSA) for 1 hour. The samples were incubated overnight at 4°Cwith primary antibodies specific for ATF4 and CHOP, diluted at 1:200. After three washes with PBS, the samples were incubated with 1:500 diluted fluorescently labeled secondary antibodies at room temperature for 1 hour, followed by three more washes. Finally, the samples were incubated in DAPI solution for 5 minutes, washed, and mounted. Observation was conducted under a fluorescence microscope, and ATF4 and CHOP expression levels were quantitatively analyzed using image analysis software.

### 2.8 RT-qPCR detection

This study employed RT-qPCR technology to assess the expression levels of miR-205-5p across different experimental cell groups, aiming to explore its role in CWP-associated nodular thyroid disease. The experiment was divided into a normal control group, a CWP group, and a treatment group, with three replicates per group to ensure data reliability and statistical significance. Total RNA was extracted from each cell group, and quantification and purity evaluation were conducted using a NanoDrop 2000. Qualifying samples were converted into cDNA using the RevertAid First Strand cDNA Synthesis Kit from Thermo Fisher. RT-qPCR was performed using the SYBR Green I fluorescent dye method with the TB Green Premix Ex Taq kit from Takara. The reaction system included 10 μL TB Green Premix Ex Taq, 1 μL forward primer, 1 μL reverse primer, 1μL cDNA template, and 7 μL nuclease-free water, with a total volume of 20μL. The cycling conditions were set as follows: initial denaturation at 95°C for 30 seconds, followed by 40 cycles of 95°C for 5 seconds and 60°Cfor 30 seconds. All experiments were conducted on an ABI 7500 Fast Real-Time PCR System.

### 2.9 Annexin V-FITC/PI dual staining flow cytometry analysis

First, 1-5×10^5 cells were collected, and the culture medium was carefully aspirated for later use. The cells were washed with phosphate-buffered saline (PBS) and digested using trypsin without EDTA to prevent interference with Annexin V binding to phosphatidylserine. The collected culture medium was then added, and the cells were gently pipetted to prepare a single-cell suspension. The cells were centrifuged at 1000 g for 5 minutes, and the supernatant was discarded. Subsequently, 1 mL of pre-chilled PBS was added to gently resuspend the cell pellet, followed by another centrifugation at 1000 g for 5 minutes, discarding the supernatant and retaining the cell pellet. Next, 195μL of Binding Buffer was added to gently resuspend the cells, followed by the addition of 5 μL Annexin V-FITC and 10 μL PI staining solution, and the mixture was gently mixed. Finally, the cells were incubated at room temperature in the dark for 10–20 minutes, with occasional gentle inversion to enhance staining effectiveness. Fluorescence intensity was detected using flow cytometry, with FITC’s maximum excitation wavelength at 488 nm and maximum emission wavelength at 525 nm.

### 2.10 Transwell assay

At 4°C, Matrigel was diluted with serum-free cell culture medium or PBS at a ratio of 1:8 (the specific dilution ratio was optimized based on the amount of MMPs produced by the cells). A total of 60 μL of the diluted Matrigel was evenly added to the bottom membrane of the upper chamber of the Transwell insert and incubated at 37°C for 3 hours to polymerize into a thin film. After incubation, the excess liquid in the upper chamber was removed, and 100 μL of serum-free medium was added, followed by a 30-minute incubation in the incubator for basal membrane hydration. The cells to be tested were cultured to the logarithmic growth phase, digested with 0.25% trypsin for 2–3 minutes, centrifuged at 1000 rpm for 3 minutes, and the supernatant was discarded. The cells were washed 1–2 times with PBS and finally suspended in serum-free medium to a concentration of 1-10×10^5/mL, adjusted according to their migration ability. In the lower chamber of a 24-well plate, 500-650 μL of medium containing 5%-10% fetal bovine serum (FBS) or chemoattractants was added. The Transwell insert was placed within this setup, and 100-200 μL of cell suspension was added to the upper chamber, followed by incubation for 12–48 hours, with the specific time adjusted based on the invasive ability of the cancer cells. At the end of the experiment, cells that migrated through the membrane and adhered to the lower side were counted directly under a microscope after staining.

### 2.11 CCK-8 assay

A 10% CCK-8 solution was added to the culture medium in each well and incubated at 37°Cwith 5% CO2 for 2–3 hours to allow the colorimetric reaction to occur. Subsequently, 100 μL of the solution from each well was transferred to a 96-well plate, and the optical density (OD) values at 450 nm wavelength were measured using a microplate reader. The results were then recorded. Relative cell viability (%) was calculated using the formula: Relative cell viability (%) = OD450’/avg (OD450C’) × 100%.

Here, OD450’ represents the absorbance value of the experimental group minus that of the blank group, while avg(OD450C’) is the average corrected absorbance value of the control group.

### 2.12 Experimental reagents and instruments

The reagents used in this study include: Fetal bovine serum (FSD500, Excell Bio, China), Penicillin-Streptomycin solution (100X, Beyotime, China), RPMI 1640 medium (Gibco, China), Trypsin (Gibco, China), Cell freezing medium (C0210B, Beyotime, China), PBS buffer (C0221A, Beyotime, China), Lipofectamine™ 3000 (Thermo Fisher, USA), Lentivirus (Envirus™, Beijing Yinggen Bio), RNA extraction and cDNA synthesis kits (Vazyme, China), Taq Pro qPCR Master Mix (Q712, Vazyme, China), BCA Protein Assay Kit (WB6501, NCM Biotech, China), Methanol (HB05, Guangzhou Chemical Reagent, China), and various antibodies (such as Anti-BAX, Anti-caspase-3, Anti-ATF4, Anti-CHOP from Abcam, USA).The instruments used include: Ultra-low temperature freezer (Haier), Optical microscope (Shanghai Optical Instrument Factory), CO2 incubator, Laminar flow cabinet (Shanghai Boxun Industrial & Medical Equipment Factory), Real-time quantitative PCR system (CFX96 Touch, Bio-Rad, USA), Western blotting system (Criterion™ Cell and Trans-Blot^®^ Cell, Bio-Rad, USA), Standard PCR machine (BIOER, China), EVOS M5000 Fluorescence Microscope (Thermo Fisher, USA), Shaker (Kylin-Bell, China), Chemiluminescence Imaging System (Shanghai JiaPeng), and Flow Cytometer (Thermo Fisher, USA). All reagents and instruments were used in strict accordance with the manufacturers’ instructions and experimental protocols to ensure the accuracy and reproducibility of the results, thereby obtaining reliable data.

### 2.13 Statistical analysis

Statistical analysis was performed using GraphPad Prism (version 9.0). All data are presented as mean ± standard deviation. One-way analysis of variance (ANOVA) was used, with p<0.05 indicating statistically significant differences between groups, and p<0.01 indicating highly statistically significant differences,>* indicates p < 0.05, ** indicates p < 0.01, *** indicates p < 0.001.

## 3 Results

### 3.1 q-PCR analysis of miR-205-5p, ATF4, and CHOP in plasma samples

**Figure 1:**
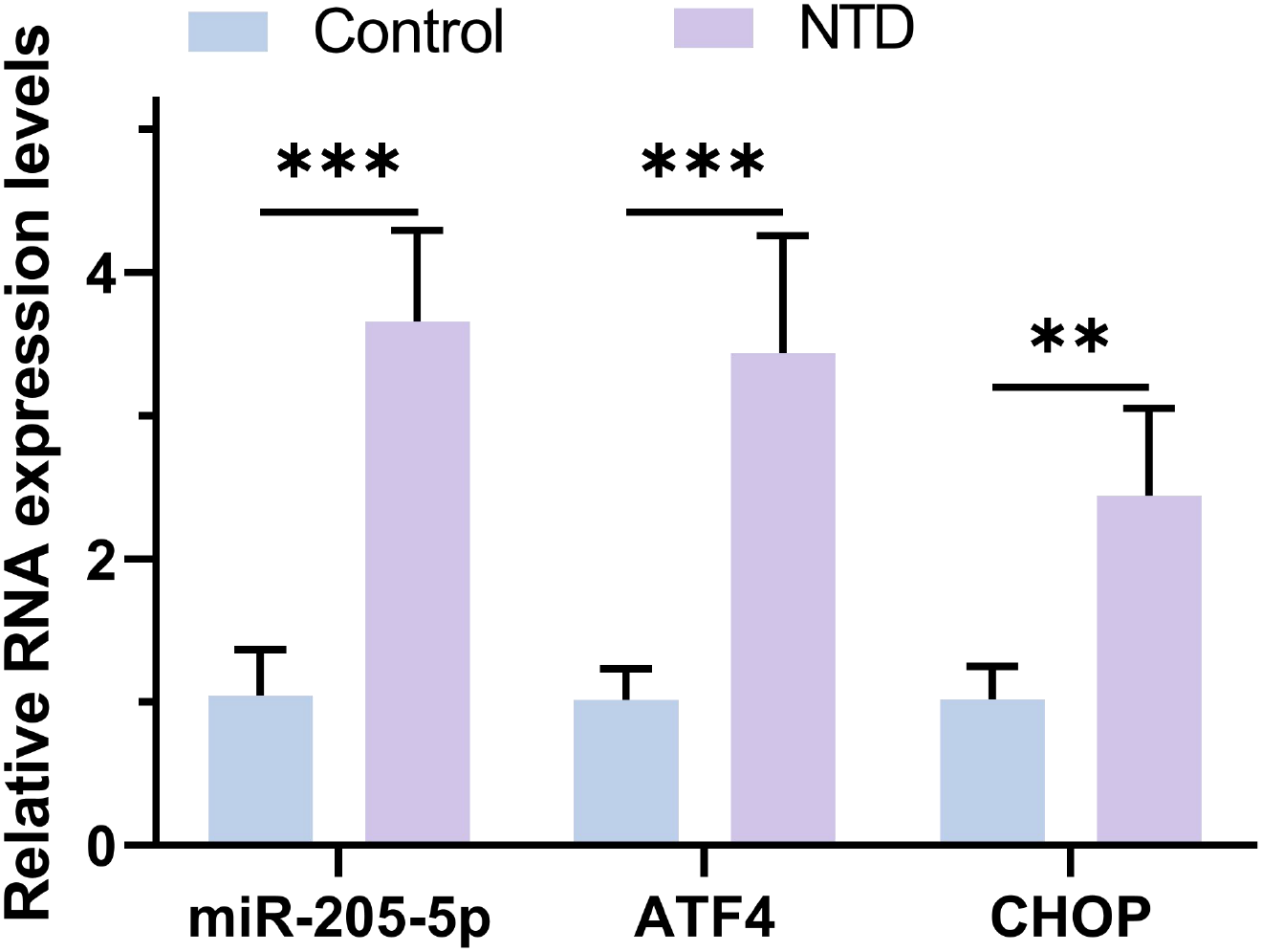
The RNA expression levels of miR-205-5p, ATF4 and CHOP were detected by RT-qPCR. **p < 0.01, ***p < 0.001.

This study conducted a comparative analysis, as illustrated in **Figure 1**, revealing that the RNA expression levels of miR-205-5p, ATF4, and CHOP in the plasma samples of the NTD patient group were significantly elevated compared to the Control normal group(S1-2). This finding not only provides additional evidence for the association between the ATF4/CHOP pathway and thyroid dysfunction but also suggests that this pathway might play a more extensive role in various types of diseases, warranting further exploration in future studies.

### 3.2 Western blot detection of ATF4/CHOP expression in bronchoalveolar lavage fluid from lung patients

**Figure 2:**
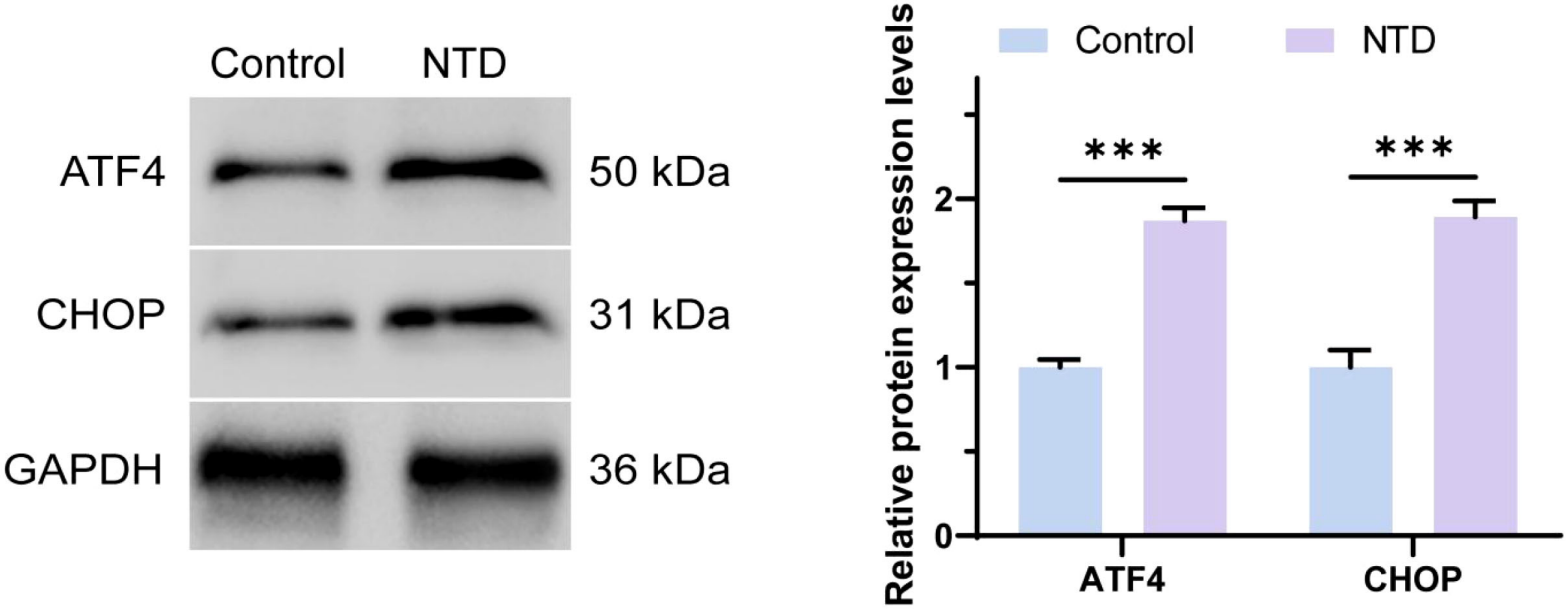
The protein expression levels of ATF4 and CHOP were detected by Western Blot. ***p < 0.001.

**Figure 3:**
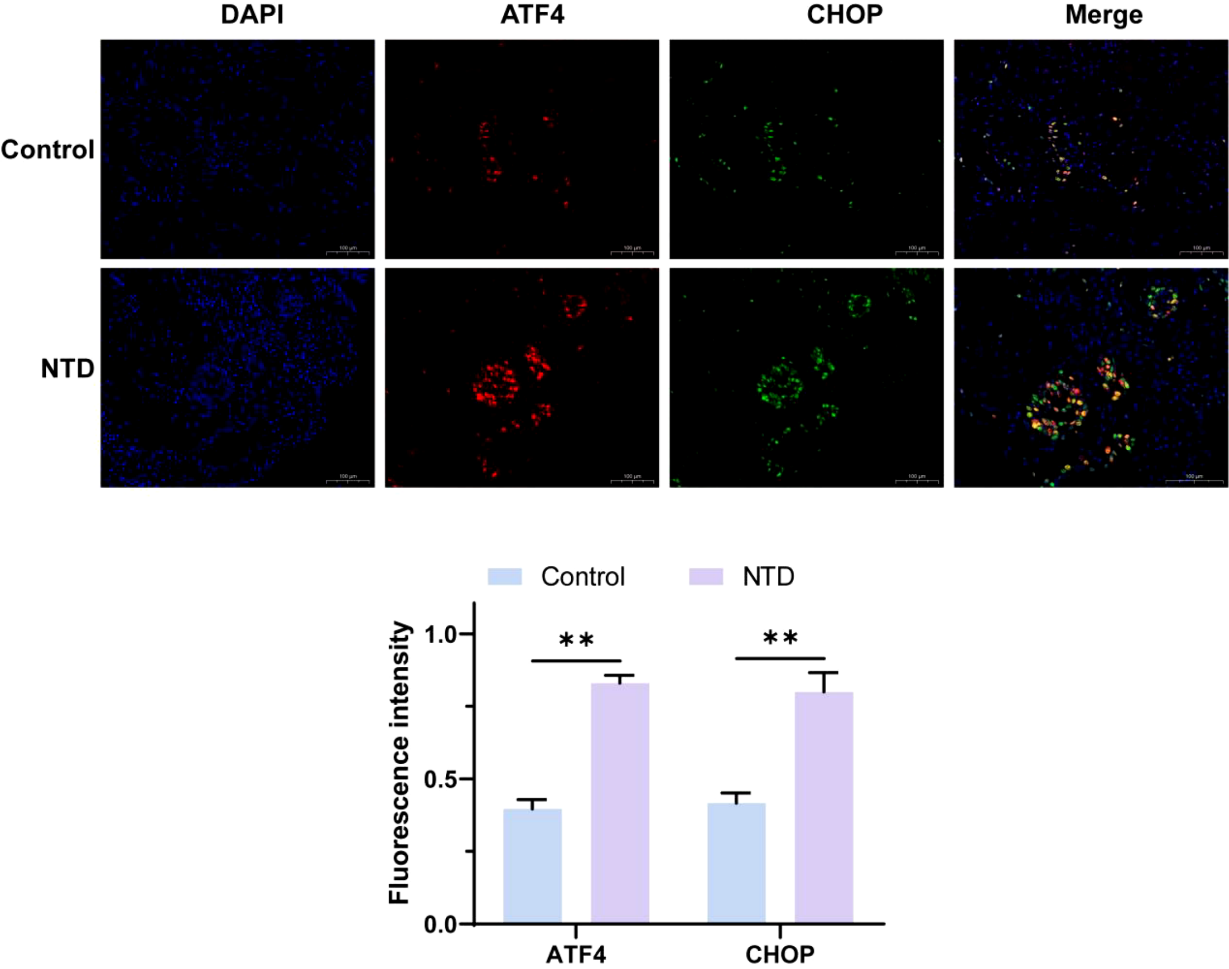
The protein expression levels of ATF4 and CHOP were detected by immunofluorescence. **p < 0.01.

**Figures 2** and **3** analyses indicate that the expression levels of ATF4 and CHOP proteins in the bronchoalveolar lavage fluid (BAL) of NTD patients are significantly higher than those in the normal control group. This is the first time that the abnormal expression of these key proteins has been directly observed in BAL samples from NTD patients. ATF4 is a stress response transcription factor whose expression is upregulated under stress conditions, while CHOP is its downstream effector, involved in apoptosis, autophagy, and metabolic adaptation. The elevation of ATF4 and CHOP in NTD patients may reflect a general stress state possibly associated with the onset and progression of NTD. As an important evaluation sample for lung diseases, changes in BAL composition can reflect the overall pathophysiological process(S3-5). Therefore, the high expression of ATF4 and CHOP in NTD patients’ BAL provides new insights into understanding their pathogenesis and suggests potential targets for the development of future NTD therapeutic strategies.

### 3.3 RT-qPCR detection of miR-205-5p expression in exosomes from 16HBE cells

**Figure 4:**
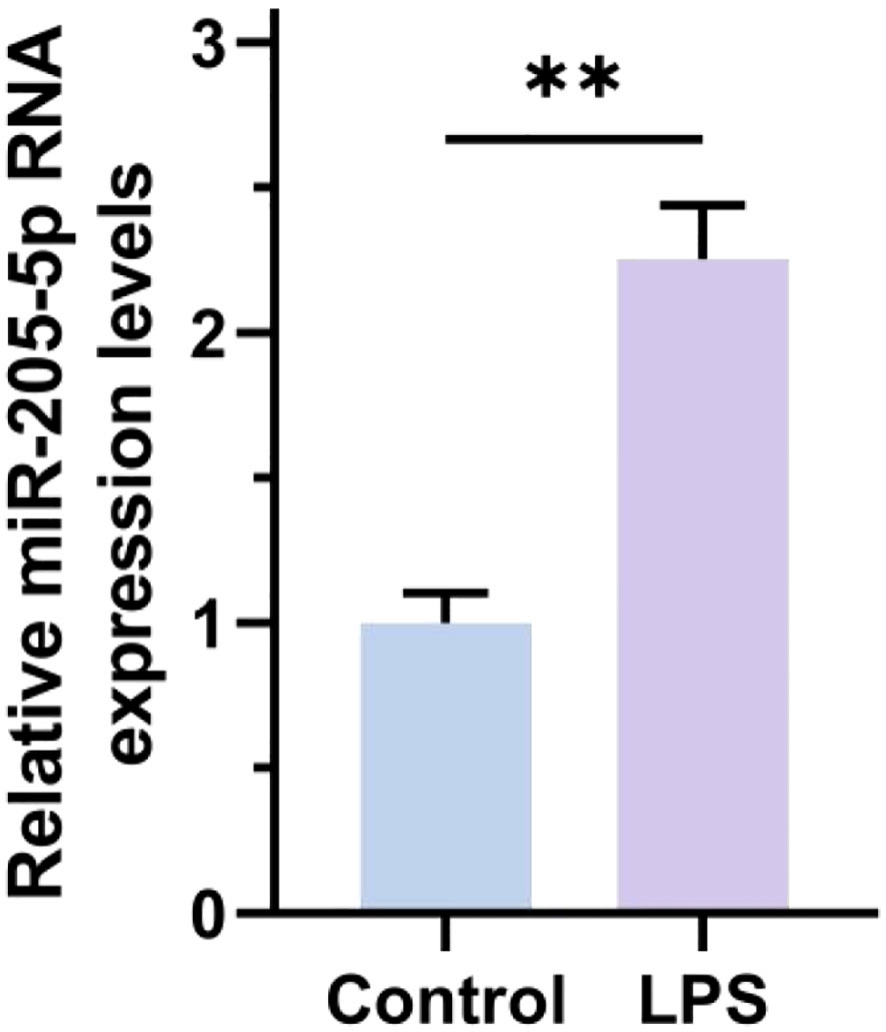
RT-qPCR detection of miR-205-5p expression in exosomes from 16HBE cells. **p < 0.01.

This study analyzed the expression levels of microRNA in exosomes secreted by 16HBE cells treated with LPS. The results showed that miR-205-5p RNA expression in the LPS group exosomes was significantly elevated compared to the normal control group. As exosomes are critical mediators for intercellular communication, carrying various bioactive molecules that influence the function of recipient cells, 16HBE cells respond to external stimuli by altering exosome composition under LPS stimulation. The high expression of miR-205-5p may be a specific response to LPS stimulation. Our previous study have demonstrated that miR-205 plays a vital role in various physiological and pathological processes, especially in inflammation-related respiratory diseases, where its expression changes are closely related to disease progression and prognosis. Therefore, the high expression of miR-205-5p in the LPS group may indicate its significant role in LPS-induced respiratory inflammation (as shown in **Figure 4**).

### 3.4 RT-qPCR detection of miR-205-5p expression in different cell groups

**Figure 5:**
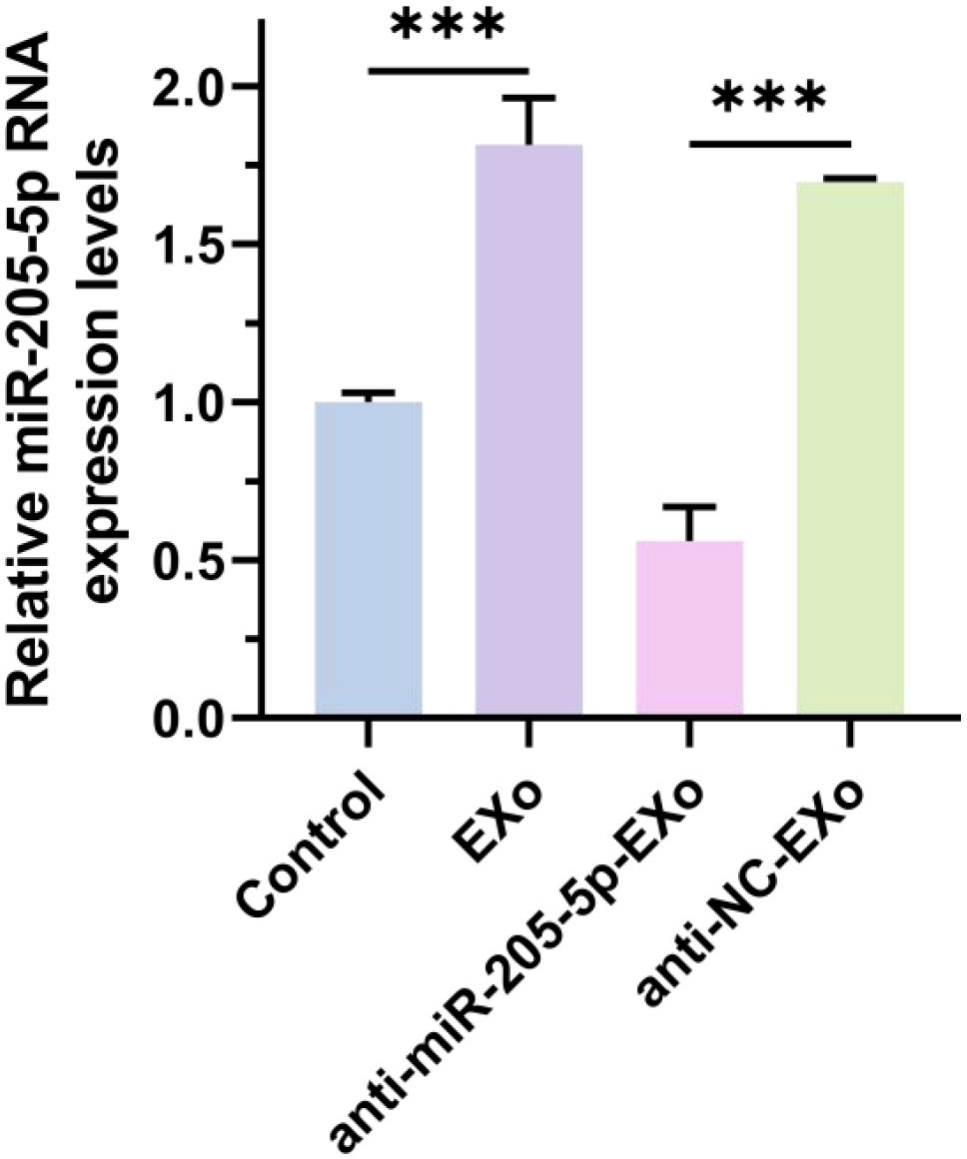
RT-qPCR detection of miR-205-5p expression in different cell groups. ***p < 0.001.

This study conducted a detailed analysis of the expression levels of miR-205-5p in thyroid papillary carcinoma cells, comparing various treatment groups. The results indicated that the group treated with exosomes (EXo) showed significantly higher expression of miR-205-5p compared to the untreated control group, suggesting that exosomes effectively deliver miR-205-5p and influence the gene expression and function of recipient cells. To verify the specific role of miR-205-5p, a specific inhibition experiment was designed, showing that the expression of this miRNA significantly decreased in exosome-treated groups with inhibited miR-205-5p (see **Figure 5**). This result confirms the specific delivery of miR-205-5p by exosomes and indicates that regulating its content can affect the miRNA expression level in thyroid papillary carcinoma cells. In summary, these data reveal the potential of exosomes to deliver miR-205-5p in thyroid papillary carcinoma cells, providing new experimental evidence for modulating this cancer’s biological behavior through exosome-mediated miRNA delivery strategies.

### 3.5 Annexin V-FITC/PI dual staining flow cytometry analysis of cell apoptosis

**Figure 6:**
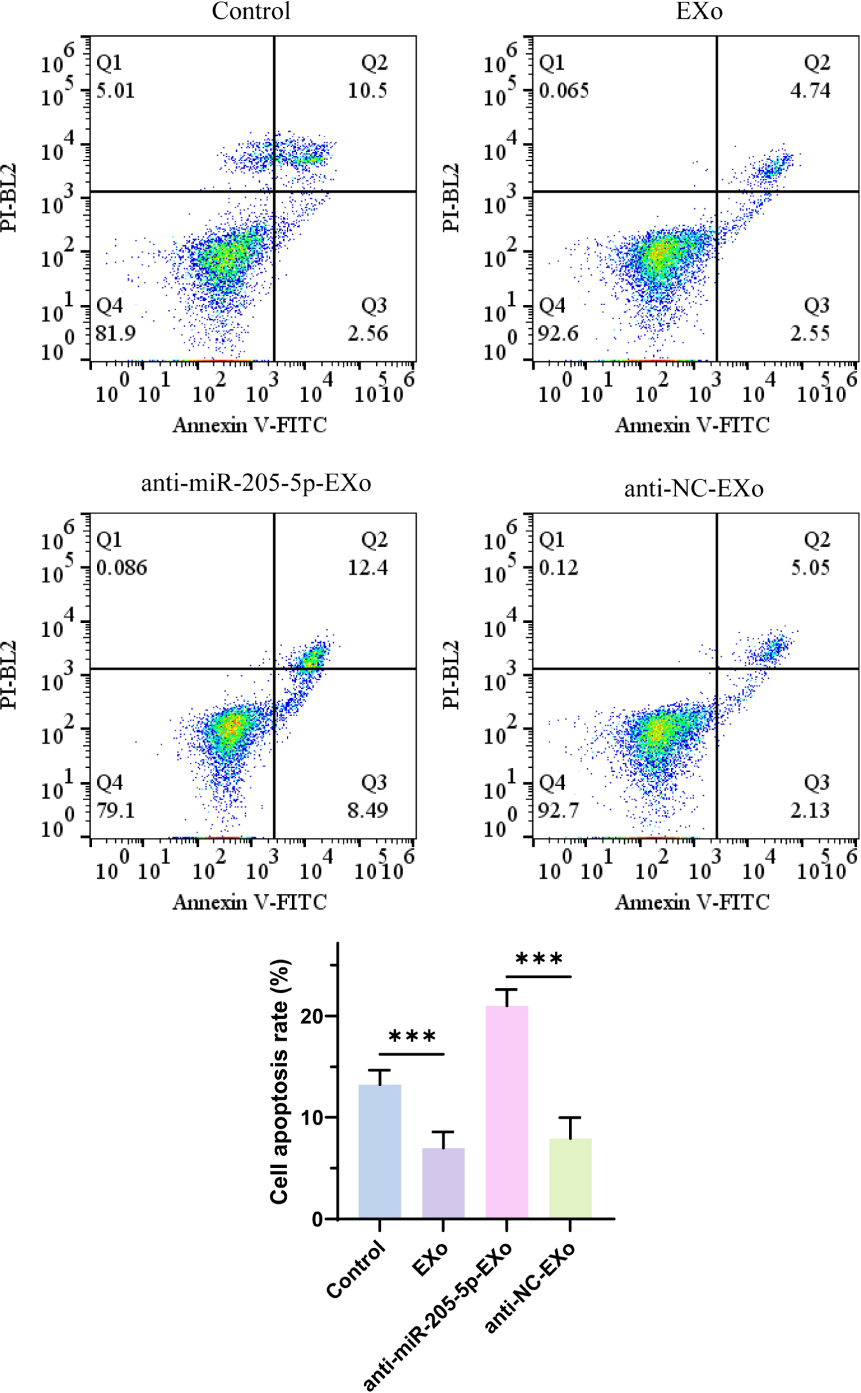
Annexin V-FITC/PI dual staining flow cytometry analysis of cell apoptosis. ***p < 0.001.

This study explored the impact of exosomes (EXo) and their cargo, miR-205-5p, on the apoptosis rate of thyroid papillary carcinoma cells. The results showed a significant reduction in the apoptosis rate in the exosome-treated group, suggesting that exosomes may carry anti-apoptotic molecules, such as miR-205-5p. To verify this hypothesis, a specific inhibition experiment was conducted, where the apoptosis rate significantly increased in the exosome-treated group with inhibited miR-205-5p (see **Figure 6**). This confirms the crucial role of miR-205-5p in inhibiting apoptosis in thyroid papillary carcinoma cells and indicates that regulating the miRNA content in exosomes can effectively influence the cell apoptosis process.

### 3.6 Western Blot analysis of BAX, caspase-3, ATF4, and CHOP expression

**Figure 7:**
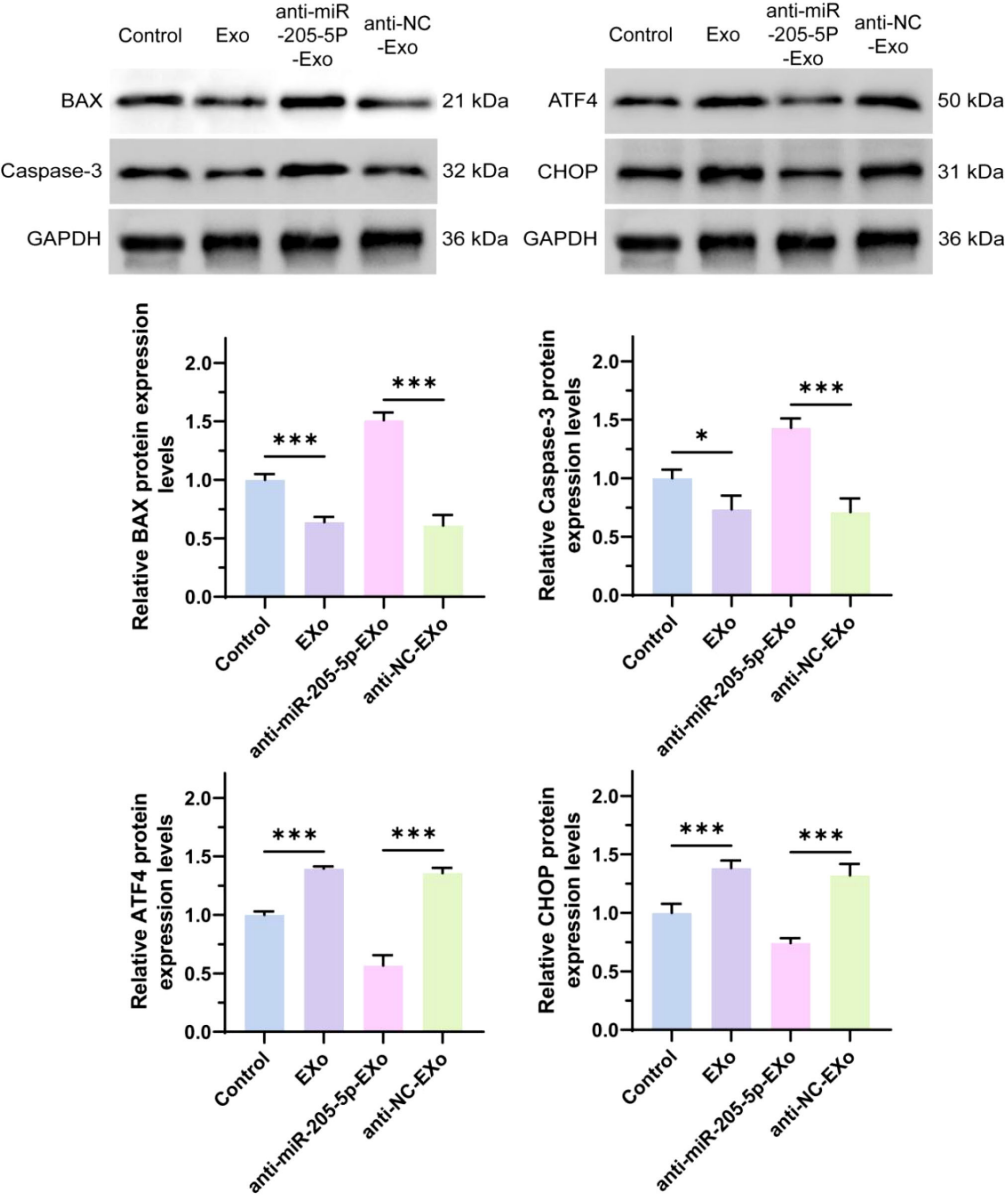
The protein expression levels of BAX, caspase-3, ATF4 and CHOP were detected by Western Blot. *p < 0.05, ***p < 0.001.

This study analyzed the effects of exosomes (EXo) and their cargo, microRNA-205-5p (miR-205-5p), on the expression of key apoptosis-related proteins—BAX, caspase-3, ATF4, and CHOP—in thyroid papillary carcinoma cells. The results showed that, compared to the untreated group, the expression of BAX and caspase-3 was significantly reduced in the EXo-treated group, whereas the expression of ATF4 and CHOP was significantly increased. This suggests that exosomes may suppress the expression of apoptosis-related proteins and promote the expression of stress response proteins via miR-205-5p. A specific inhibition experiment confirmed that, compared to the non-specific control group, inhibiting miR-205-5p led to significantly increased expression of BAX and caspase-3, while ATF4 and CHOP expression decreased (as shown in **Figure 7**). This result confirms the key role of miR-205-5p in regulating these protein expressions and indicates that modulating the miR-205-5p content in exosomes can effectively influence the apoptosis process in thyroid papillary carcinoma cells.

### 3.7 Transwell assay for evaluating cell invasion and migration abilities

**Figure 8:**
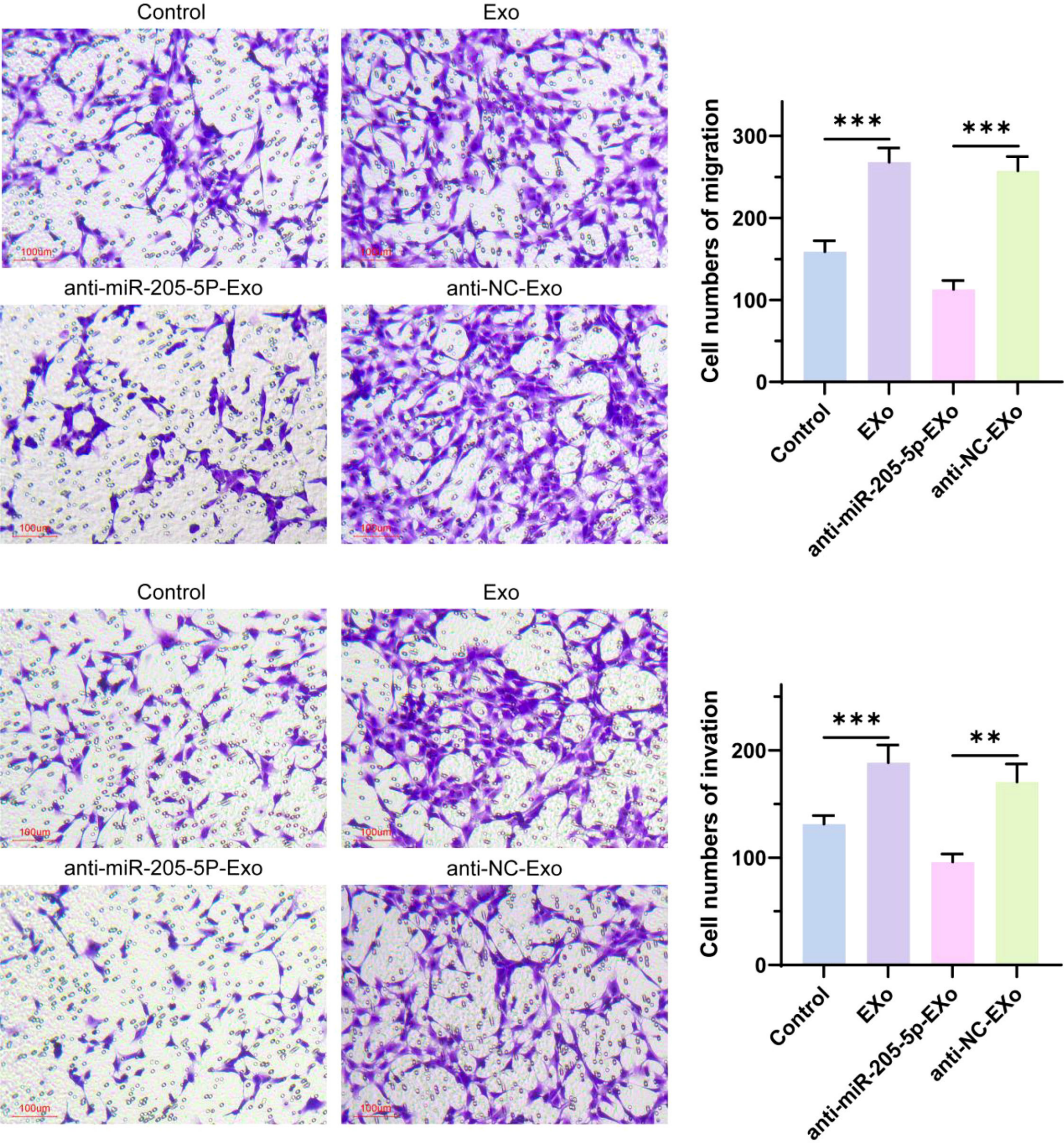
The migration and invasion ability of cells were detected by Transwell. **p < 0.01, ***p < 0.001.

This study assessed the effects of exosomes (EXo) and their cargo, microRNA-205-5p (miR-205-5p), on the migration and invasion capabilities of thyroid papillary carcinoma cells. The results demonstrated that the EXo-treated group exhibited significantly enhanced cell migration and invasion compared to the untreated control group, suggesting that exosomes may carry molecules that promote tumor migration and invasion. To validate this hypothesis, a specific inhibition experiment was conducted, revealing that inhibiting miR-205-5p resulted in significantly reduced migration and invasion capabilities of thyroid papillary carcinoma cells compared to the non-specific control group (see **Figure 8**). This finding confirms the critical role of miR-205-5p in promoting cell migration and invasion and indicates that modulating the miR-205-5p content in exosomes can effectively suppress the malignant biological behavior of thyroid papillary carcinoma cells.

### 3.8 CCK-8 assay for cell proliferation activity

**Figure 9:**
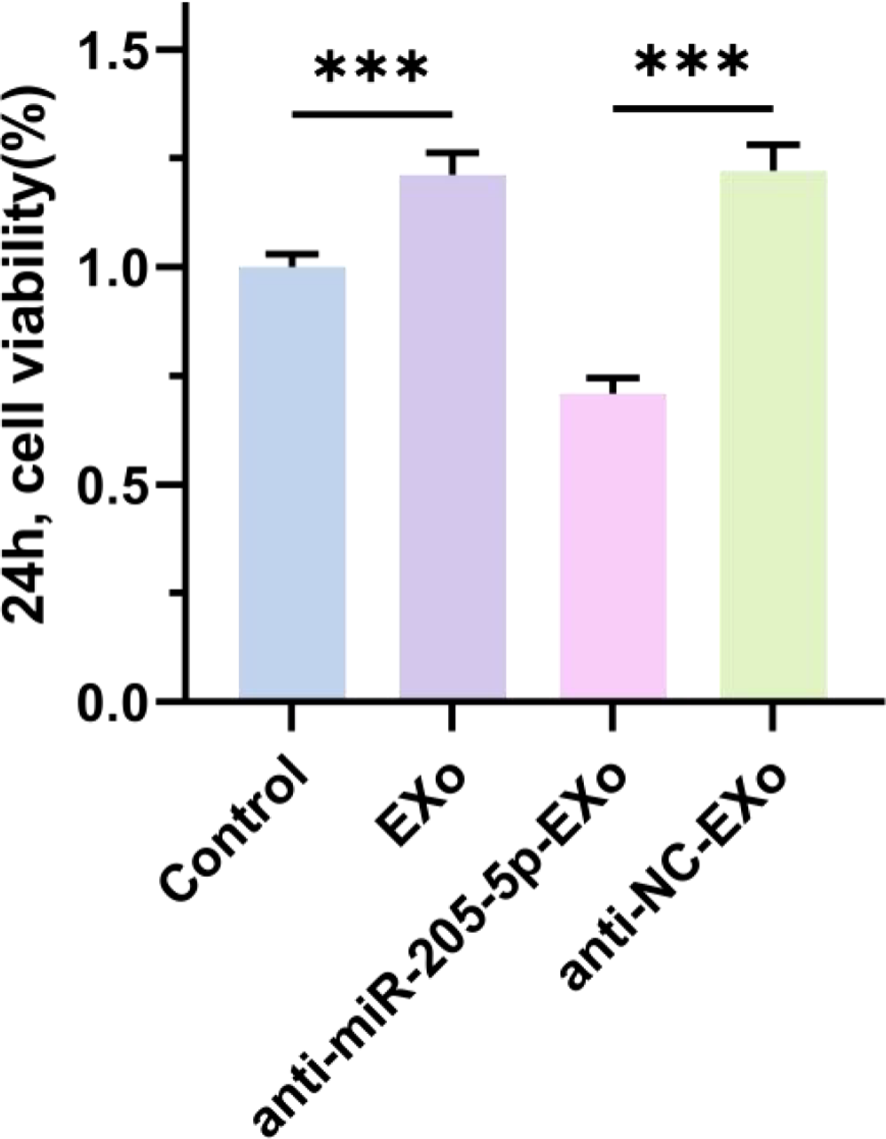
The cell viability was detected by CCK8. ***p < 0.001.

This study evaluated the impact of exosomes (EXo) and their cargo, microRNA-205-5p (miR-205-5p), on the proliferation capability of thyroid papillary carcinoma cells. The results showed that the proliferation capability of cells in the EXo group was significantly enhanced compared to the untreated control group, indicating that exosomes may carry molecules that promote cell proliferation. To verify this hypothesis, a specific inhibition experiment was conducted, revealing that the proliferation capability significantly decreased in the group with inhibited miR-205-5p. This further confirms the critical role of miR-205-5p in promoting the proliferation of thyroid papillary carcinoma cells and suggests that regulating the miR-205-5p content in exosomes can effectively inhibit cell proliferation (see **Figure 9**).

### 3.9 miR-205-5p regulates endoplasmic reticulum stress by inhibiting the ATF4/CHOP pathway

**Figure 10:**
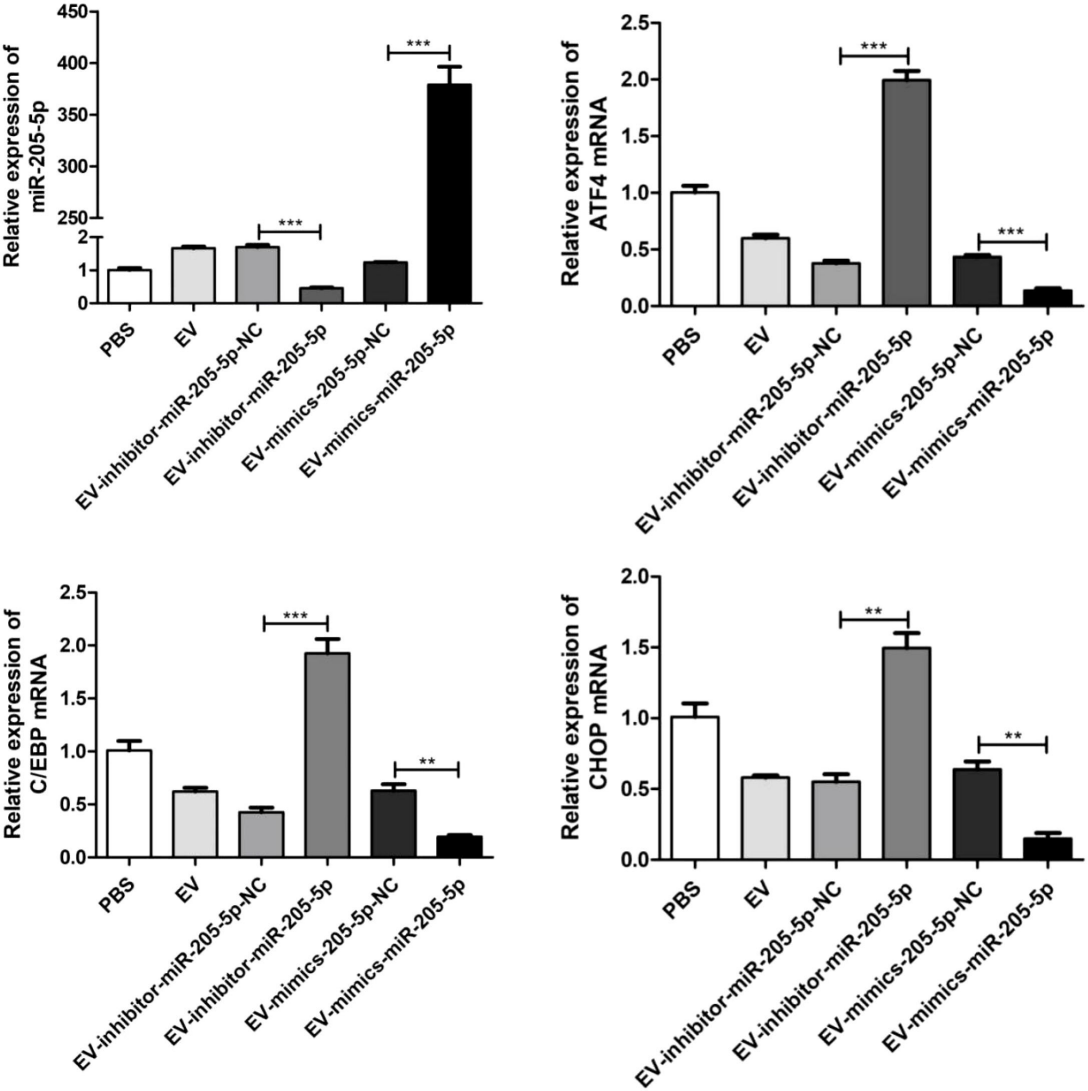
q-PCR technique was used to investigate the effects of miR-205-5p on the expression of ATF4, CHOP, and C/EBP genes. **p < 0.01, ***p < 0.001.

This experiment utilized Q-PCR technology to investigate the effects of miR-205-5p on the expression of ATF4, CHOP, and C/EBP genes. The experiment was divided into five groups: the EV group (transfection of an empty vector as the baseline control), the EV-inhibitor-miR-205-5p-NC group (transfection of a negative control inhibitor to exclude non-specific effects), the EV-mimics-205-5p-NC group (transfection of a negative control mimic to exclude operational interference), the EV-inhibitor-miR-205-5p group (transfection of a specific inhibitor to inhibit miR-205-5p expression), and the EV-mimics-miR-205-5p group (transfection of a specific mimic to overexpress miR-205-5p). The results showed that compared with the EV control group, the expression levels of ATF4, CHOP, and C/EBP in the miR-205-5p inhibitor group (EV-inhibitor-miR-205-5p) were significantly upregulated (increased approximately 2.5 times, 2.0 times, and 1.5 times respectively), while the expression levels of these genes in the miR-205-5p mimic group (EV-mimics-miR-205-5p) were significantly downregulated (reduced to approximately 0.5 times, 0.4 times, and 0.6 times respectively). The negative control groups (EV-inhibitor-miR-205-5p-NC and EV-mimics-205-5p-NC) showed no significant difference compared with the EV group, indicating good experimental specificity. These results confirmed that miR-205-5p has a negative regulatory effect on ATF4, CHOP, and C/EBP, suggesting that it may exert biological functions by inhibiting the endoplasmic reticulum stress pathway. Further, we conducted Western Blot experiments to detect the protein expression levels of ATF4 and CHOP in different treatment groups (**Figure 10**). The experimental results showed that the regulatory effect of miR-205-5p on the protein expression levels of ATF4 and CHOP was observed in samples with suppressed miR-205-5p expression, and the protein expression levels of ATF4 and CHOP showed an upward trend (see **Supplementary Figure S6**).

## 4 Discussion

Coal Workers’ Pneumoconiosis (CWP) is an occupational lung disease caused by prolonged inhalation of coal dust, primarily affecting coal miners. The hallmark pathological features of CWP include lung tissue fibrosis and nodule formation (12–16). Despite ongoing global coal mining activities, the incidence of CWP remains relatively high in certain regions, particularly in developing countries (14, 17–20). Studies indicate that CWP not only results in impaired lung function but is also associated with various comorbidities, including cardiovascular disease, chronic obstructive pulmonary disease, and lung cancer (21–29). Furthermore, recent research has highlighted the potential risk of thyroid dysfunction in patients with CWP, especially the development of nodular thyroid disease (NTD) (30).

Research both domestically and internationally has explored the association between CWP and other diseases (14–16). For instance, a study by Zhao F et al. (2021) demonstrated a correlation between the incidence of CWP among coal miners and various endocrine disorders, such as thyroid diseases (30, 31). Kulkybaev GA et al. (31) found that prolonged exposure to coal dust might impact the endocrine system of coal miners, potentially leading to thyroid dysfunction. These findings suggest that CWP could affect thyroid health through complex pathological mechanisms, though the specific molecular mechanisms remain unclear. Against this backdrop, microRNAs (miRNAs) have gained attention as critical intracellular regulatory molecules. miRNAs play pivotal roles in numerous biological processes, including cell proliferation, apoptosis, and inflammatory responses (32–35). Our previous study has established that specific miRNAs are crucial in the pathogenesis of tumors and metabolic diseases. Notably, miR-205 has been implicated in promoting tumor growth in breast and prostate cancers, and it is also linked to the progression of thyroid cancer (36–38).

However, the role of miR-205-5p in CWP-associated thyroid diseases has not been systematically studied. Given the aforementioned context, this study aimed to explore the expression changes of miR-205-5p in CWP cases and its relationship with the ATF4/CHOP signaling pathway, analyzing its potential role in nodular thyroid disease. This research not only bridges the knowledge gap between CWP and NTD but also provides new insights into the mechanisms underlying CWP-associated endocrine diseases, laying the groundwork for the development of future clinical interventions and therapeutic strategies. For the first time, this study investigates the role of pulmonary-derived exosomal microRNA-205-5p (miR-205-5p) in the development of nodular thyroid disease (NTD) associated with CWP, offering new insights. By comparing 30 CWP cases to 30 healthy controls, we found significantly elevated levels of ATF4 and CHOP in the plasma and bronchoalveolar lavage fluid (BAL) of NTD patients, suggesting that the ATF4/CHOP signaling pathway could play a crucial role in the pathogenesis of NTD.

ATF4, as a stress response transcription factor, has been confirmed by numerous studies to play a crucial role under various disease conditions. Previous research has shown that ATF4 promotes apoptosis during endoplasmic reticulum stress by upregulating CHOP expression (39–41). For instance, Zong et al. (40) discovered that the specific induction of CHOP contributes to the responsiveness of thyroid cancer cells to proteasome inhibitors; ATF4 and Nrf2 regulate the molecular mechanisms of CHOP induction in thyroid cancer cells through proteasome inhibition. This finding provides a new perspective on understanding the role of the ATF4/CHOP pathway in thyroid diseases. Our results align with these findings, emphasizing the central role of the ATF4/CHOP pathway in maintaining cellular homeostasis and regulating thyroid dysfunction.

In our cellular experiments, we observed a significant increase in miR-205-5p levels in the exosomes from LPS-treated 16HBE cells, suggesting its important role in respiratory inflammatory responses. This finding is consistent with previous studies, which have reported that miR-205 plays a significant role in various physiological and pathological processes, especially in the initiation and progression of tumors (42–44). For example, miR-205 is considered a critical regulatory factor in both breast cancer (45) and prostate cancer (46). Our study further indicates that miR-205-5p, through exosomes, participates in the development of thyroid diseases in CWP cases, suggesting that the interaction between exosomes and miR-205-5p might represent a novel pathological mechanism.

In experiments involving thyroid papillary carcinoma cells from NTD patients, exosome treatment significantly increased the expression levels of miR-205-5p and inhibited the expression of apoptosis-related proteins such as BAX and caspase-3. These results suggest that miR-205-5p might have an anti-apoptotic effect through exosomes, thereby promoting cancer cell survival. This finding is in line with existing research, indicating that miR-205-5p may play a promotive role in tumor growth and metastasis. Some researchers (44) have found that miR-205 is crucial in promoting cell proliferation and inhibiting apoptosis in lung cancer.

Although this study provides new insights into the role of miR-205-5p in CWP-associated thyroid diseases, there are some limitations. First, the sample size is relatively small and limited to coal miners from a specific region, which may affect the generalizability of the results. Future studies should aim to expand the sample size to include patients from diverse regions and occupational backgrounds to further validate our findings. Additionally, the single-center design of this study may introduce regional and population-specific biases. A multicenter study involving coal miners from different geographic and environmental settings would enhance the robustness and applicability of the results.

Another limitation is that our research primarily focused on the expression of miR-205-5p and its function in cell models, with limited exploration of its specific signaling mechanisms and downstream target genes. Future research should delve into the specific interactions between miR-205-5p and the ATF4/CHOP pathway, as well as its role in other types of thyroid diseases and tumors. Furthermore, the lack of longitudinal data limits our ability to establish causal relationships between miR-205-5p expression and disease progression. Prospective cohort studies with long-term follow-up are needed to address this gap. Moreover, the widespread role of the ATF4/CHOP signaling pathway in various diseases suggests that this pathway could be a new therapeutic target for thyroid diseases. We plan to explore potential intervention strategies targeting this pathway, such as using small molecule drugs or specific RNA interference techniques, to assess their effectiveness in regulating thyroid function and treating related diseases.

Not only did this study confirm via Q-PCR experiments that miR-205-5p significantly suppresses the expression of ATF4, CHOP, and C/EBP, but it also provides novel molecular evidence for elucidating the regulatory role of miR-205-5p in cellular stress responses. Experimental results demonstrated that inhibition of miR-205-5p led to a 2.5-fold increase in ATF4 expression, a 2.0-fold increase in CHOP expression, and a 1.5-fold increase in C/EBP expression, whereas overexpression of miR-205-5p reduced the expression levels of these genes to 50%-60% of the control group. This dose-dependent regulatory relationship strongly indicates that miR-205-5p may serve as a critical negative regulator of the endoplasmic reticulum stress (ERS) pathway. Notably, ATF4 and CHOP, as core components of the PERK/eIF2α/ATF4/CHOP signaling pathway, play pivotal roles in modulating cell apoptosis through their expression changes. Our findings further substantiate that miR-205-5p may maintain cellular homeostasis by regulating the ATF4/CHOP/C/EBP axis. Additionally, Western Blot analysis confirmed the regulatory effect of miR-205-5p on the protein expression levels of ATF4 and CHOP. In samples where miR-205-5p expression was inhibited, the protein expression levels of ATF4 and CHOP exhibited an upward trend. These results not only enhance our understanding of the miRNA regulatory network but also provide potential molecular targets for developing therapeutic strategies for ERS-related diseases. Future investigations could explore the specific mechanisms of miR-205-5p under various pathological conditions and its clinical application prospects.

Overall, this study not only provides new evidence for the relationship between CWP and nodular thyroid disease but also lays an important biological foundation for exploring the role of miR-205-5p in pulmonary and thyroid diseases. We hope this research will advance the understanding of the pathological mechanisms of CWP and provide a theoretical basis for future clinical treatment and prevention strategies. As research into the functions of exosomes and miRNAs progresses, more precise therapeutic strategies may be developed in the future, improving the quality of life for CWP cases and offering more effective management options for thyroid diseases.

## 5 Conclusion

This study explores the role of lung-derived exosomal miR-205-5p in coal workers’ pneumoconiosis (CWP) patients with concurrent nodular thyroid disease (NTD). The results show a significant increase in miR-205-5p within the exosomes of CWP cases, which is associated with elevated levels of ATF4 and CHOP, suggesting that the ATF4/CHOP signaling pathway may play a crucial role in CWP-related NTD. Additionally, the study found that miR-205-5p expression is increased in the exosomes from LPS-treated 16HBE cells, indicating its regulatory role in respiratory inflammation. In thyroid papillary carcinoma cells, exosome treatment elevated the expression of miR-205-5p and inhibited apoptosis-related proteins BAX and caspase-3, suggesting that miR-205-5p may promote cancer cell survival.

In summary, this research uncovers the potential mechanism of exosomal miR-205-5p in thyroid diseases among CWP cases and highlights its potential as a biomarker and therapeutic target. It offers a new perspective for understanding the molecular connection between CWP and NTD, providing a scientific basis for future clinical applications. Subsequent studies should further validate the specific role and application potential of miR-205-5p.

## Data Availability

The raw data supporting the conclusions of this article will be made available by the authors, without undue reservation.
