# Supplementary material for: Involvement of miR-205-5p in mediating the development of nodular thyroid disease associated with coal worker’s pneumoconiosis via pulmonary extracellular vesicles
Source: Front Endocrinol (Lausanne). 2025 Jul 4;16:1528330. doi: 10.3389/fendo.2025.1528330 (PMC12271885; doi:10.3389/fendo.2025.1528330)
Supplement: Supplementary file 1 [file DataSheet1.docx]

# **Supplementary Material**

**Section 1: Supplementary Tables**

Table S1 Conditional sequence of qPCR reaction

| Phase | Reactivity | Cycle number | Temperature | Time |
| --- | --- | --- | --- | --- |
| Stage 1 | Predegeneration | Reps：1 | 95℃ | 30 sec |
| Stage 2 | Cyclic reaction | Reps：40 | 95℃ | 10 sec |
|  |  |  | 60℃ | 10 sec |
| Stage 2 | Melting curve | Reps：1 | 95℃ | 15 sec |
|  |  |  | 65℃ | 60 sec |
|  |  |  | 95℃ | 15 sec |

Table S2 Primers sequence

| Primers | Sequence (5’→3’) | |
| --- | --- | --- |
| GIMAP4  ATF4  CHOP  miR-205-5p  GAPDH | Forward  Reverse  Forward  Reverse  Forward  Reverse  Forward  Reverse  Forward  Reverse | ACCAGGCATTTTCGACACAG  TGTTTGCTTCTGGATCTCCTC  CCCGCCCACAGATGTAGTTT  CGCTCGTTAAATCGCTTCCC  GCTCAGGAGGAAGAGGAGGA  TCCTGCTTGAGCCGTTCATT  GTCCTTCATTCCACCGGAGT  GCTCCATGCCTCCTGAACTT  ACCACAGTCCATGCCATCAC  TCACCACCCTGTTGCTGTA |

# **Section 2: Supplementary Figures**


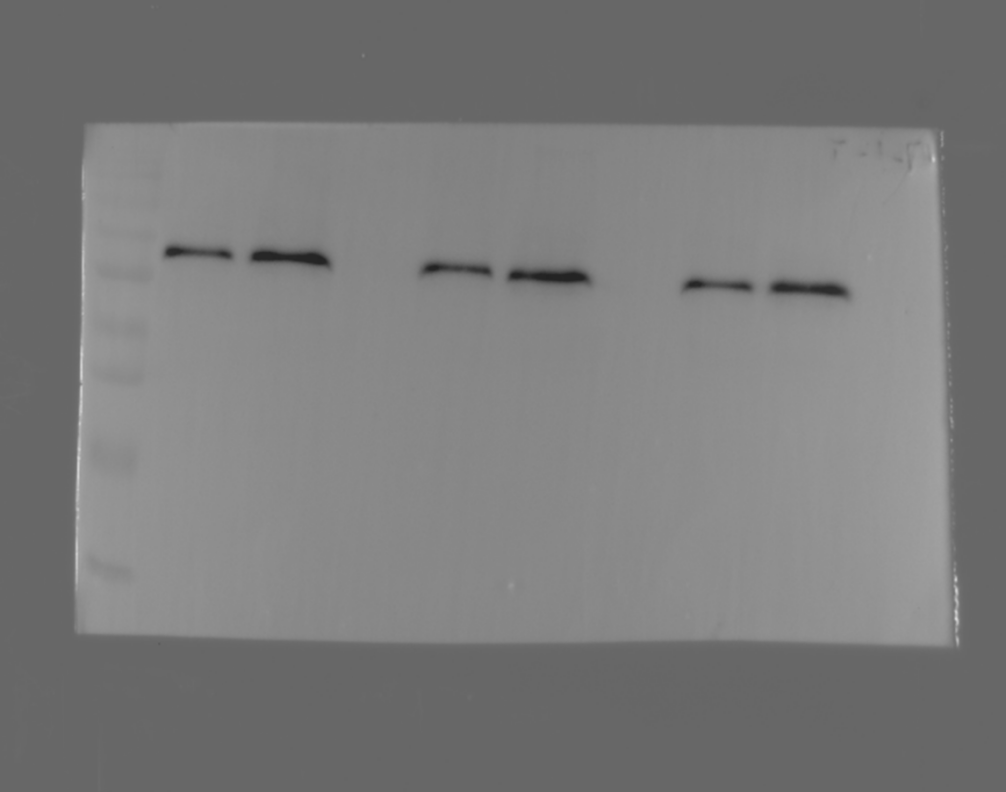


Figure **S1**  ATF4-50kDa-westernblot detection of the entire cut film
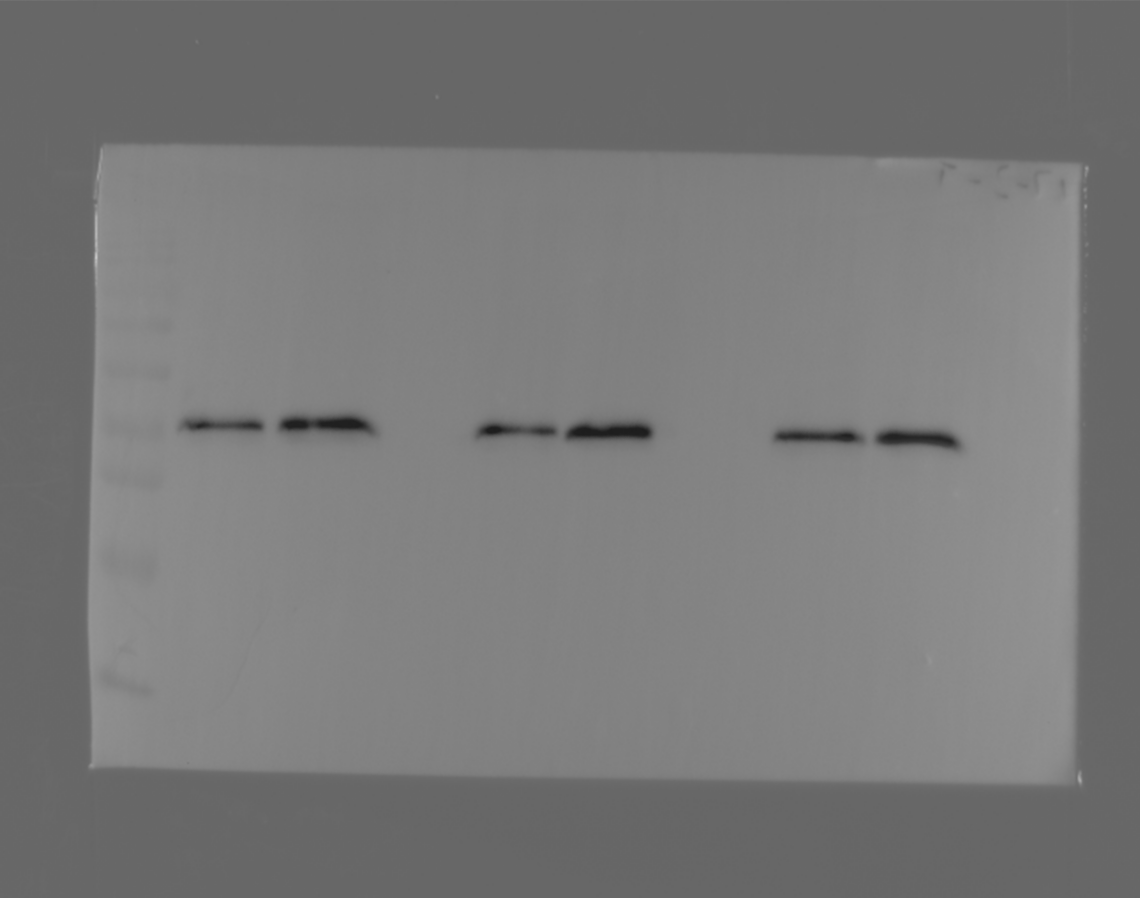


Figure **S2** CHOP-31kDa-westernblot detection of the entire cut film
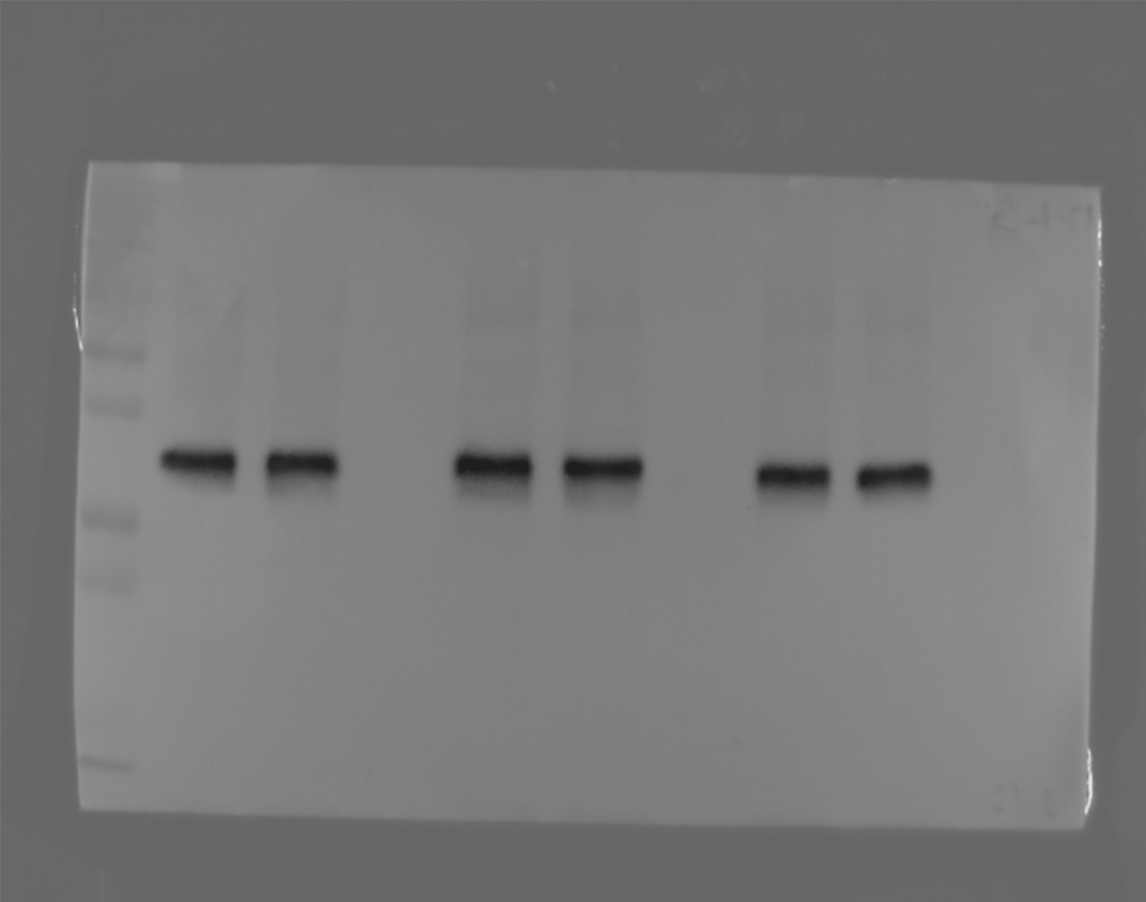


Figure **S3** GAPDH-36kDa-westernblot detects the entire cut film


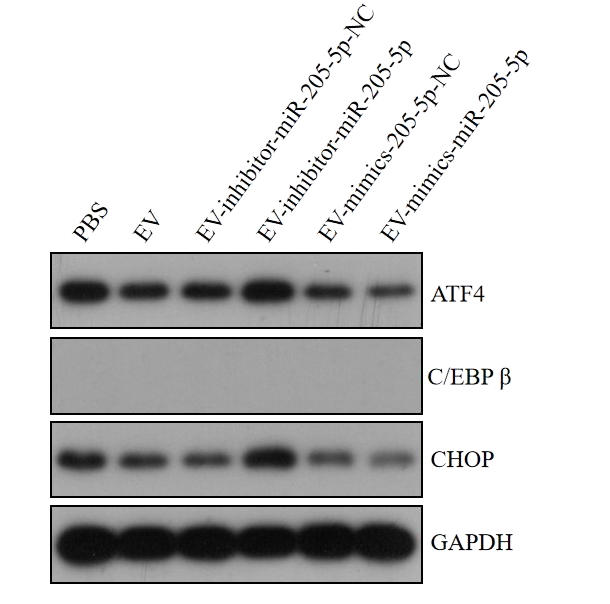


Figure **S4** The regulatory effect of miR-205-5p on the expression of ATF4 and CHOP proteins

**(Note: Western Blot was used to detect the protein expression levels of ATF4 and CHOP in different treatment groups, with GAPDH serving as the internal reference protein for normalization.)**
